# Comparison of Tegoprazan and Lansoprazole in Patients With Erosive Esophagitis up to 4 Weeks: A Multi‐Center, Randomized, Double‐Blind, Active‐Comparator Phase 4 Trial

**DOI:** 10.1111/nmo.14969

**Published:** 2024-11-25

**Authors:** Cheol Min Shin, Suck Chei Choi, Jin Woong Cho, Seung Young Kim, Ok Jae Lee, Do Hoon Kim, Yu Kyung Cho, Ju Yup Lee, Sang Kil Lee, Jeong Eun Shin, Gwang Ha Kim, Seon‐Young Park, Su Jin Hong, Hye‐Kyung Jung, Sang Jin Lee, Young Hoon Youn, Seong Woo Jeon, In Kyung Sung, Moo In Park, Oh Young Lee

**Affiliations:** ^1^ Department of Internal Medicine Seoul National University Bundang Hospital Seongnam Korea; ^2^ Department of Gastroenterology, School of Medicine Wonkwang University Iksan Korea; ^3^ Department of Internal Medicine Presbyterian Medical Center Jeonju Korea; ^4^ Department of Gastroenterology Korea University Ansan Hospital Ansan Korea; ^5^ Department of Internal Medicine Gyeongsang National University Hospital Jinju Korea; ^6^ Department of Gastroenterology, Asan Medical Center University of Ulsan College of Medicine Seoul Korea; ^7^ Department of Internal Medicine The Catholic University of Korea Seoul St. Mary's Hospital Seoul Korea; ^8^ Department of Internal Medicine Keimyung University Dongsan Hospital Daegu Korea; ^9^ Department of Gastroenterology, Severance Hospital Yonsei University College of Medicine Seoul Korea; ^10^ Department of Gastroenterology Dankook University Hospital Cheonan Korea; ^11^ Department of Internal Medicine Pusan National University College of Medicine, and Biomedical Research Institute, Pusan National University Hospital Busan Korea; ^12^ Department of Gastroenterology Chonnam National University Hospital Gwangju Korea; ^13^ Department of Internal Medicine Soonchunhyang University Bucheon Hospital Bucheon Korea; ^14^ Department of Internal Medicine College of Medicine, Ewha Womans University Seoul Korea; ^15^ Department of Internal Medicine, Gangneung Asan Hospital University of Ulsan College of Medicine Gangneung Korea; ^16^ Department of Gastroenterology, Gangnam Severance Hospital Yonsei University College of Medicine Seoul Korea; ^17^ Department of Gastroenterology Kyungpook National University Chilgok Hospital Daegu Korea; ^18^ Department of Internal Medicine Konkuk University Medical Center Seoul Korea; ^19^ Department of Gastroenterology Kosin University Gospel Hospital Busan Korea; ^20^ Department of Internal Medicine Hanyang University College of Medicine Seoul Korea

**Keywords:** erosive esophagitis, lansoprazole, potassium competitive acid blocker, proton pump inhibitor, tegoprazan

## Abstract

**Background:**

The aims of this study were to confirm the non‐inferiority of tegoprazan to lansoprazole up to week 4 in patients with erosive esophagitis (EE) and to evaluate its effectiveness in rapid mucosal healing and symptom relief at week 2.

**Methods:**

In this multi‐center, randomized, double‐blind, active‐comparator non‐inferiority trial, 218 patients with endoscopically confirmed EE (Los Angeles Classification Grades A–D) were randomly allocated to either the tegoprazan (50 mg) or lansoprazole (30 mg) group. The primary endpoint was the cumulative proportion of patients with healed EE up to week 4, as confirmed through endoscopy. The proportion of patients with healed EE at week 2 was also evaluated. Furthermore, *CYP2C19* genotypes, symptoms, safety, and tolerability were assessed.

**Key Results:**

In the full‐analysis set, 103 and 109 participants in the tegoprazan and lansoprazole groups, respectively, were analyzed. The cumulative healing rates up to week 4 were 95.2% (98/103) and 86.2% (94/109) (difference [95% confidence interval], 8.91 [1.22–16.59]; *p* < 0.0001 for non‐inferiority and 0.0266 for superiority), while those at week 2 were 88.4% (91/103) and 82.6% (90/109) (5.78 [−3.66–15.22], *p* = 0.0005 for non‐inferiority) for tegoprazan and lansoprazole, respectively. Tegoprazan showed consistent healing rates regardless of *CYP2C19* genotypes.

**Conclusions and Inferences:**

Tegoprazan was superior to lansoprazole in the treatment of EE up to 4 weeks. Further studies are necessary to confirm these findings and clarify the superiority of tegoprazan, especially in the treatment of severe EE.

**Trial Registration:**

ClinicalTrials.gov identifier: NCT05267743

## Graphical abstract

Tegoprazan 50 mg demonstrated non‐inferior efficacy to lansoprazole 30 mg in healing erosive esophagitis at 2 weeks and showed superior efficacy up to 4 weeks. It may be effective in faster healing and symptom relief, especially in cases with severe erosive esophagitis.

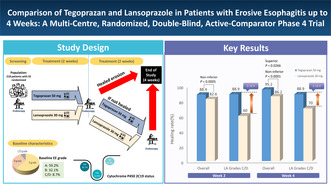

**Summary**

- Tegoprazan 50 mg showed non‐inferior efficacy in healing erosive esophagitis (EE) compared to lansoprazole 30 mg at 2 weeks and up to 4 weeks when administered once daily.
- Moreover, tegoprazan 50 mg was superior to lansoprazole 30 mg in the treatment of EE up to 4 weeks.
- Tegoprazan 50 mg may be effective in faster EE healing and symptom relief. It may be more beneficial for patients with severe EE. However, additional clinical trials are necessary to confirm these findings.

## 1 Introduction

In recent decades, the prevalence of gastroesophageal reflux disease (GERD), defined as the abnormal reflux of gastric contents into the esophagus, has increased worldwide [1, 2, 3]. In Korea, the prevalence of GERD is 3.4%–6.4% [1, 4, 5]. Proton pump inhibitors (PPIs) are widely used for the treatment of various acid‐related disorders, including GERD and peptic ulcer disease [6]. Recently, potassium competitive acid blockers (P‐CABs) such as vonoprazan, fexuprazan, and tegoprazan have been marketed as replacements for PPIs in clinical practice [7, 8]. Of note, P‐CABs display rapid onset and have a longer half‐life than PPIs [9]. Moreover, the time of dosing of P‐CABs is flexible, unlike that of most PPIs.

In previous studies, P‐CABs revealed excellent efficacy in treating erosive esophagitis (EE) [10, 11]. In a recent meta‐analysis, 8‐week administration of P‐CABs showed results similar to those of PPIs in individuals with EE. Although most studies have compared vonoprazan with PPIs, one study compared tegoprazan with esomeprazole [12]. In this study, the up‐to‐week‐8 mucosal healing rate of tegoprazan was 96%, whereas that of esomeprazole was approximately 93%, demonstrating the non‐inferiority of tegoprazan in the treatment of EE. A recent clinical trial also showed the non‐inferior efficacy of fexuprazan to esomeprazole in treating EE [8]. Based on these results, recent guidelines have proposed that the effect of P‐CABs was comparable to that of PPIs in the initial treatment of patients with GERD [13, 14].

Regarding the optimal initial treatment period of GERD using PPIs, most guidelines currently recommend an initial treatment duration of 4–8 weeks [13, 15]. However, P‐CABs allow rapid and stable inhibition of gastric acid secretion with the first dose [16]. To date, most studies on the effects of P‐CABs in EE have predominantly evaluated esophageal mucosal healing after 8 weeks as the primary outcome; studies on the effects after 4 weeks of treatment as the primary outcome are rare. Therefore, we hypothesized that using P‐CABs instead of PPIs shortens the initial treatment period of EE.

In this study, we first confirmed the non‐inferiority of tegoprazan to lansoprazole in subjects with EE up to week 4. We also performed a pilot experiment to evaluate whether tegoprazan was effective in the rapid healing of EE and rapid relief of GERD symptoms at 2 weeks.

## 2 Materials and Methods

### 2.1 Study Participants

The study was designed as a multi‐center, randomized, double‐blind, non‐inferiority trial. Overall, 218 individuals aged 18–80 years with endoscopically confirmed EE were randomly allocated to either the tegoprazan or lansoprazole group. The study was conducted at 20 sites in South Korea from February 16, 2021, to May 25, 2022. The clinical protocol was approved by the institutional review boards of each institute and followed the principles of the Declaration of Helsinki. All participants signed an informed consent form before inclusion in the study.

Exclusion criteria were as follows: (1) inability to undergo upper endoscopy; (2) positive *Helicobacter pylori* test; (3) inability to maintain a daily symptom diary; (4) presence of peptic stricture, esophageal varices, Barrett's esophagus, eosinophilic esophagitis, active peptic ulcer, or bleeding during screening endoscopy; (5) previous surgery or plans to undergo a surgery that could affect gastric acid secretion (i.e., upper gastrectomy and vagotomy); (6) diagnosis of functional dyspepsia, primary esophageal motility disorder, irritable bowel syndrome, or inflammatory bowel disease within 3 months before enrollment; (7) history of malignancy within 3 years before enrollment; (8) pregnancy or lactation; (9) use of any PPI 14 d before enrollment; (10) use of any drugs related to GERD treatment (i.e., histamine 2 receptor antagonists, mucoprotectants, prokinetics, antacids) more than twice within 1 week before enrollment; (11) use of human immunodeficiency virus protease inhibitors (i.e., atazanavir, nelfinavir) or medications containing rilpivirine; (12) clinically significant abnormal values in screening tests including alanine aminotransferase, aspartate aminotransferase, alkaline phosphatase, γ‐glutamyl transpeptidase, and total bilirubin levels more than twice the upper reference limit (UNL) or blood urea nitrogen and creatinine levels more than 1.5 times the UNL; (13) history of Zollinger–Ellison syndrome or gastric acid hypersecretion disorders; (14) permission for continuous use of non‐steroidal anti‐inflammatory drugs during the study period; low‐dose aspirin (no more than 100 mg/day) to prevent cardiovascular or cerebrovascular events; (15) clinically significant hepatic, renal, cardiovascular, respiratory, endocrine, or central nervous system disorders; and (16) known hypersensitivity to PPIs and P‐CABs.

Following a screening period, patients were randomized 1:1 to receive either oral tegoprazan or oral lansoprazole once daily. This study group was assigned by central enrollment, and the Interactive Web Response System was used for randomization. Information on randomization was securely stored, and it could be accessed by authorized personnel only. To ensure that the study was double‐blinded, a double‐dummy method using matched tegoprazan and lansoprazole was adopted. All medications were provided in sealed boxes and supplied by a medication supervisor to ensure blind allocation.

### 2.2 Study Protocol

The entire study protocol is summarized in Figure S1. On the first day of the screening period, the demographics and other baseline characteristics of the study participants, including medical history, comorbidities, medication history, and concomitant medications, were collected. Physical examination and laboratory (complete blood count, serum chemistry, and urinalysis), pregnancy, electrocardiogram, and *H. pylori* tests were also performed. Participants recorded the occurrence of diurnal or nocturnal heartburn in their diary before going to bed (once a day) during the treatment period.

At week 2 (or upon early termination) and week 4, physical examination and laboratory tests were performed. Additionally, vital signs (including electrocardiogram), adverse events, concomitant medication, diary records, and treatment compliance were checked.

Endoscopy was performed by principal investigators with at least 3 years of endoscopy experience at the start of the screening period and at week 2 (or upon early termination) and week 4. The severity of esophagitis was defined based on the endoscopic findings according to the Los Angeles (LA) classification grade from A to D [17]. Healed EE was defined as the absence of esophageal mucosal erosions or ulcers on esophagogastroduodenoscopy. The study protocol was terminated 2 weeks after administering the study drug if the results of endoscopy indicated mucosal healing. If EE was not healed at week 2, the study drug was administered for an additional 2 weeks, followed by a second endoscopy at week 4.

Changes in symptoms were assessed using the Reflux Disease Questionnaire (RDQ), which is a 12‐item self‐administered questionnaire designed to assess the frequency and severity of heartburn, acid regurgitation, and dyspepsia [18]. Changes in the mean RDQ score were evaluated from baseline to week 2 or 4.

Finally, an intention‐to‐treat analysis (full‐analysis set, FAS) was conducted on 103 participants in the tegoprazan group and 109 participants in the lansoprazole group.

### 2.3 Outcome Evaluation

The primary endpoint was the cumulative proportion of patients with healed EE confirmed via endoscopy up to week 4. Secondary endpoints included the (1) proportion of patients with healed EE confirmed via endoscopy at week 2, (2) cumulative proportion of patients with healed EE at weeks 2 and 4 according to *CYP2C19* genotypes, (3) complete resolution rates of heartburn, and (4) changes in RDQ scores.

Safety was assessed by physical examination and analysis of adverse events, laboratory test values, and vital signs. Information on concomitant medications and frequency and severity of adverse events was collected throughout the study. A treatment‐emergent adverse event (TEAE) was defined as an adverse event that occurred during treatment, representing a change from baseline. All TEAEs were graded based on severity as severe, moderate, or mild by the investigator. A drug‐related TEAE was an adverse event that was deemed by the investigator as possibly related or related to the study drug. A serious TEAE was defined as an adverse event that could cause death, hospitalization, disability, congenital anomaly, or a life‐threatening condition.

### 2.4 Statistical Analysis

The results of previous studies showed that the proportion of patients with healed EE up to week 4 was 96.6% for vonoprazan 20 mg and 92.5% for lansoprazole 30 mg [10]. Consequently, the proportion was assumed to be 94.6% (50% fraction of difference between vonoprazan and lansoprazole) for tegoprazan 50 mg and 92.5% for lansoprazole 30 mg. With this assumption, the sample size in the present study was calculated to be 87 participants per treatment group. An *α*‐error of 0.025 (one‐sided) and power ≥ 90% was applied to detect the non‐inferiority of tegoprazan to lansoprazole with a non‐inferiority margin of 10%. Considering a dropout rate of 20%, 109 participants (= 87/[1 − 0.2]) were required for each treatment group.

For the primary endpoint, the proportion of patients with healed EE up to week 4 was calculated in the FAS and per‐protocol set (PPS) population. The non‐inferiority of tegoprazan to lansoprazole was tested by comparing the lower bound of two‐sided 95% confidence intervals (CIs) of the difference in healing rate, with a non‐inferiority margin of 10%. Similar analyses were performed for the proportion of patients with healed EE up to week 2. Unpaired *t*‐test or Wilcoxon rank‐sum tests were performed to compare continuous variables including the changes in symptom scores between groups, and the results were presented as the mean and standard deviation (SD). Other categorical variables were expressed as the number (%) and compared using the *χ* ^2^ test or Fisher's exact test, as appropriate. All analyses were performed using the SAS Enterprise Guide, version 9.4 (SAS Institute Inc.), and *p* < 0.05 was considered statistically significant.

## 3 Results

### 3.1 Baseline Characteristics of Study Participants

**FIGURE 1:**
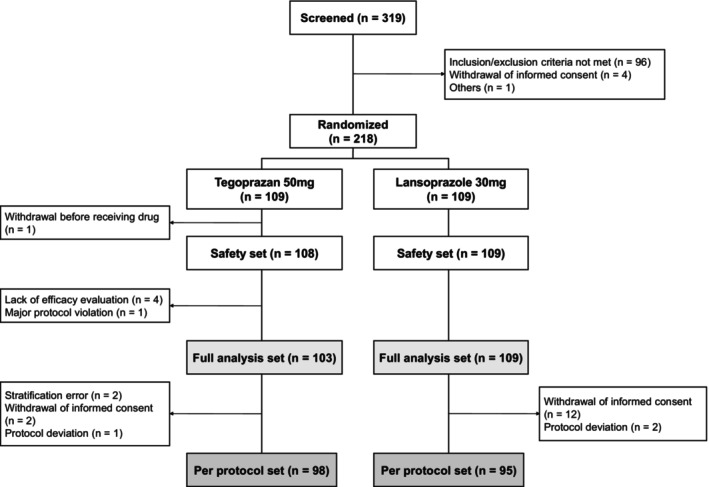
Randomization protocol and patient dispositions.

Out of the 319 patients with EE screened in the study, 218 eligible participants were randomized to receive either tegoprazan 50 mg (*n* = 109) or lansoprazole 30 mg (*n* = 109). The FAS included 103 patients in the tegoprazan group (one patient withdrew before receiving the drug, no efficacy data were collected for four patients, and one patient made a major protocol violation) and 109 patients in the lansoprazole group. The PPS included 98 and 95 patients in the tegoprazan and lansoprazole group, respectively. The details of each treatment group are summarized in the flowchart in Figure 1.

Table 1 presents the demographics and baseline characteristics of the study participants (randomized set). Most variables of the two groups were comparable, except for the dyspepsia severity score, with the lansoprazole group having more severe dyspeptic symptoms than the tegoprazan group (1.18 vs. 1.55, *p* = 0.0297). Notably, most Korean patients with GERD had mild EE (LA grade A or B), whereas only 9% (19 of 218) of the study participants had severe EE.

**TABLE 1:** Subject demographics and baseline characteristics (randomized set).

	Tegoprazan 50 mg (*n* = 109)	Lansoprazole 30 mg (*n* = 109)	*p*
**Men, *n* (%)**	61 (56.0)	75 (68.8)	0.0503^a^
**Age, mean (SD), years**	53.97 (15.68)	52.06 (15.28)	0.3639^b^
**Current smoker, *n* (%)**	17 (15.6)	21 (19.3)	0.4752^a^
**Alcohol use, *n* (%)**	40 (36.7)	43 (39.5)	0.6756^a^
**RDQ, mean (SD)**			
Heartburn			
Severity score	1.56 (1.19)	1.54 (1.02)	0.9029^b^
Frequency score	1.57 (1.15)	1.70 (1.11)	0.3688^b^
Dyspepsia			
Severity score	1.18 (1.12)	1.55 (1.37)	0.0297^b^
Frequency score	1.27 (1.20)	1.59 (1.35)	0.0648^b^
Regurgitation			
Severity score	1.24 (1.24)	1.45 (1.22)	0.2164^b^
Frequency score	1.31 (1.25)	1.58 (1.30)	0.1190^b^
**Baseline erosive esophagitis, *n* (%)**			0.7458^a^
LA grade A	65 (59.6)	64 (58.7)	
LA grade B	35 (32.1)	35 (32.1)	
LA grade C	8 (7.3)	10 (9.2)	
LA grade D	1 (0.9)	0 (0.00)	
**Cytochrome P450 2C19 status, *n* (%)** ^c^			0.0851^a^
Extensive metabolizer	40 (36.7)	43 (39.5)	
Intermediate metabolizer	49 (45.0)	48 (44.0)	
Poor metabolizer	14 (12.8)	18 (16.5)	—

Abbreviations: LA, Los Angeles; RDQ, Reflux Disease Questionnaire; SD, Standard deviation.

^a^ *χ* ^2^ test.

^b^ Unpaired *t*‐test.

^c^ Six patients in the tegoprazan 50 mg group were excluded due to missing results.

### 3.2 Healing Rate of EE

In the FAS, the cumulative EE healing rates up to week 4 (primary endpoint) were 95.2% (98/103) and 86.2% (94/109) for the tegoprazan and lansoprazole group, respectively (difference [95% CI]: 8.91 [1.22–16.59], *p* < 0.0001 in the *χ* ^2^ test for non‐inferiority, 0.0266 in the *χ* ^2^ test for superiority, Table 2). The healing rates at week 2 were 88.4% (91/103) and 82.6% (90/109) for the tegoprazan and lansoprazole group, respectively (difference [95% CI]: 5.78 [−3.66–15.22], *p* = 0.0005 in the *χ* ^2^ test for non‐inferiority). Similar results were observed in the PPS; tegoprazan was non‐inferior to lansoprazole up to week 4 as well as at week 2 (Table 2).

**TABLE 2:** Healing rate (%) of erosive esophagitis in the two groups.

	Tegoprazan 50 mg	Lansoprazole 30 mg
**Up to week 4**		
Full‐analysis set		
Healing rate, % (*n*/*N*)	95.2 (98/103)	86.2 (94/109)
Difference (95% CI) _(tegoprazan − lansoprazole)_	8.91 (1.22, 16.59)^a^	
Non‐inferiority *p*‐value	< 0.0001^a^	
Superiority *p*‐value	0.0266^b^	
Per‐protocol set		
Healing rate, % (*n*/*N*)	96.9 (95/98)	96.8 (92/95)
Difference (95% CI) _(tegoprazan − lansoprazole)_	0.10 (−6.78, 6.98)^c^	
Non‐inferiority *p*‐value	0.0020^c^	
Superiority *p*‐value	1.0000^b^	
**At week 2**		
Full‐analysis set		
Healing rate, % (*n*/*N*)	88.4 (91/103)	82.6 (90/109)
Difference (95% CI) _(tegoprazan − lansoprazole)_	5.78 (−3.66, 15.22)^a^	
Non‐inferiority *p*‐value	0.0005^a^	
Superiority *p*‐value	0.2338^b^	
Per‐protocol set		
Healing rate, % (*n*/*N*)	89.8 (88/98)	92.6 (88/95)
Difference (95% CI) _(tegoprazan − lansoprazole)_	−2.84 (−10.81, 5.13)^a^	
Non‐inferiority *p*‐value	0.0390^a^	
Superiority *p*‐value	0.4871^b^	

Abbreviation: CI, confidence interval.

^a^ *χ* ^2^ test for non‐inferiority/Wald CI.

^b^ *χ* ^2^ test or Fisher's exact test.

^c^ Score test for non‐inferiority/Exact CI.

### 3.3 Response According to Cytochrome P450 2C19 Genotypes and Severity of EE

**FIGURE 2:**
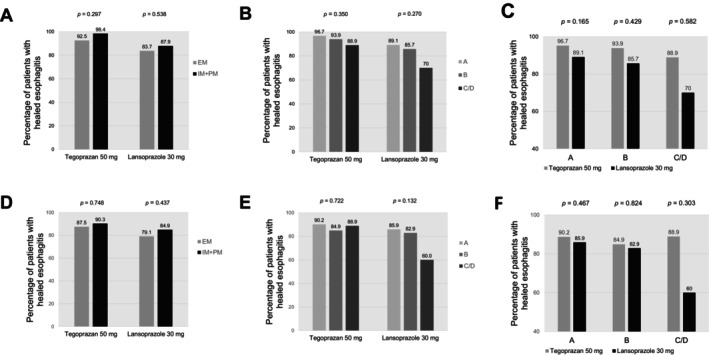
Subgroup analysis of mucosal healing rates up to week 4 (A–C) and at week 2 (D–F, full‐analysis set). Comparing mucosal healing rates by *CYP2C19* genotypes (A, D) and the severity of esophagitis (Los Angeles grade; B, E) in each treatment group, and comparing mucosal healing rates by treatment group (C, F) based on the severity of esophagitis. *p*‐values were calculated using the unpaired *t*‐test. EM, extensive metabolizer; IM, intermediate metabolizer; PM, poor metabolizer.

Figure 2 shows that tegoprazan resulted in consistent healing rates regardless of *CYP2C19* genotypes (92.5% and 98.4% in extensive metabolizers [EM] and intermediate or poor metabolizers [IM + PM], respectively, up to week 4 [Figure 2A]; 87.5% and 90.3% in EM and IM + PM, respectively, at week 2 [Figure 2D], all *p* > 0.05). Contrary to our expectations, no significant differences were observed in cumulative healing rates by *CYP2C19* genotypes even in the lansoprazole group (83.7% and 87.9% in EM and IM + PM, respectively, up to week 4 [Figure 2A]; 79.1% and 84.9% in EM and IM + PM, respectively, at week 2 [Figure 2D], all *p* > 0.05).

Notably, tegoprazan maintained higher healing rates in patients with severe EE (LA grade C or D) (96.7%, 93.9%, and 88.9% in LA grades A, B, and C/D, respectively, up to week 4 [Figure 2B]; 90.2%, 84.9%, and 88.9% in LA grades A, B, and C/D, respectively, at week 2 [Figure 2E], *p* > 0.05). In contrast, lansoprazole treatment resulted in lower healing rates in patients with severe EE compared with those in patients with mild EE (LA grade A or B), albeit with no statistical significance (89.1%, 85.7%, and 70.0% in LA grades A, B, and C/D, respectively, up to week 4 [Figure 2B]; 85.9%, 82.9%, and 60.0% in LA grades A, B, and C/D, respectively, at week 2 [Figure 2E], *p* > 0.05). When comparing mucosal healing rates by treatment group based on the severity of EE, tegoprazan showed a higher healing rate than lansoprazole in patients with severe EE (LA grade C/D: 88.9% vs. 70.0% up to week 4 [Figure 2C] and 88.9% vs. 60.0% at week 2 [Figure 2F]); however, the difference in healing rate was not statistically significant.

### 3.4 Symptom Response

**FIGURE 3:**
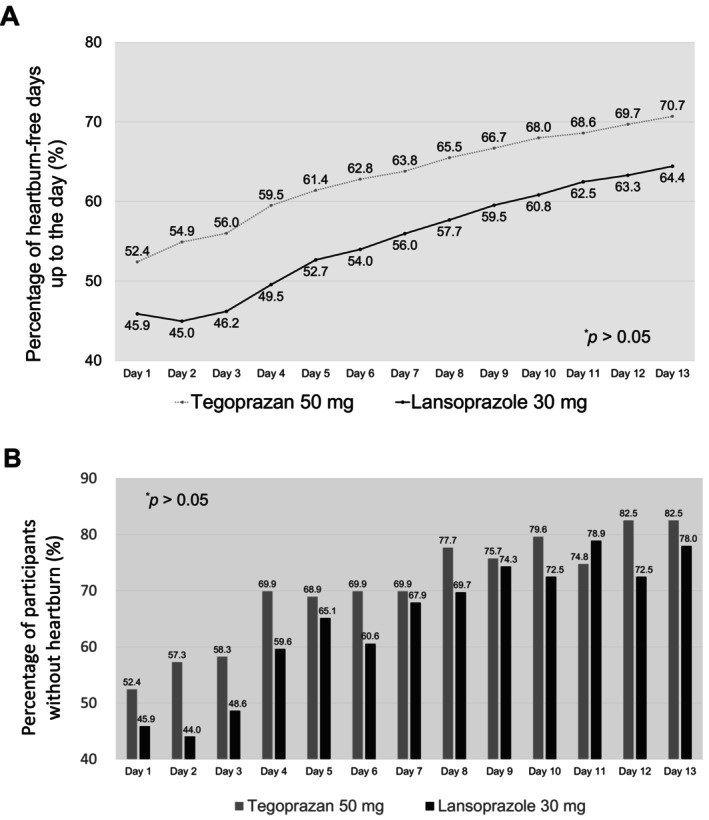
Proportion of days without heartburn calculated daily (A) and daily proportions of patients without heartburn (B) during the first 2 weeks after drug administration in the tegoprazan and lansoprazole groups.

We observed that the daily proportion of participants without heartburn and proportion of days without heartburn calculated daily were higher in the tegoprazan group than in the lansoprazole group, albeit with no statistical significance (Figure 3). Furthermore, the changes in the RDQ score at week 4 were not different between the two groups (Table S1).

### 3.5 Tolerability and Safety

The rates of adverse events in the tegoprazan group were comparable with those in the lansoprazole group (Table S2). No participants reported serious drug‐related TEAEs in either group. Only 1 (0.93%) patient in the tegoprazan group showed mild elevations in the levels of liver enzymes, which were not related to the study drug.

## 4 Discussion

This study is the first to investigate the 4‐week efficacy of tegoprazan in treating EE as the primary endpoint. In this study, tegoprazan 50 mg was confirmed to be non‐inferior to lansoprazole 30 mg in terms of mucosal healing at week 2 as well as up to week 4 in patients with EE (Table 2). Moreover, although this study was not originally designed as a superiority trial, tegoprazan was concluded to be superior because it showed a significantly higher mucosal healing rate than lansoprazole up to week 4 (95.2% vs. 86.2%, *p* = 0.0266, Table 2). These findings provide evidence that 4 weeks of treatment with P‐CABs may be sufficient as the initial treatment of EE [19]. Moreover, the perceived superiority of tegoprazan over lansoprazole in this study may have been influenced by EE severity and the comparator antisecretory drug. In the first Korean trial, tegoprazan administered at 50 or 100 mg once daily was non‐inferior to esomeprazole 40 mg in achieving notable healing rates of EE at both week 4 (90.3% vs. 88.5%) and week 8 (99.1% vs. 99.1%) [12]. In the present study, the superior efficacy of tegoprazan at 4 weeks could be attributed either to a higher proportion of patients with severe EE (percentage of the patients with LA grade C/D: 8.7% [19/218] in the present study vs. 4.3% [13/300] in the study by Lee et al. [12]) or to the lower‐ and shorter‐lasting antisecretory activity of lansoprazole compared to esomeprazole [20].

In addition, tegoprazan maintained high mucosal healing rates regardless of the severity of EE and *CYP2C19* genotype (Figure 2). One of the drawbacks of PPIs is that they undergo liver catabolism mainly via cytochrome P450 2C19 (CYP2C19), which shows genetic polymorphisms, leading to interindividual variations in their pharmacokinetic and pharmacodynamic profiles [21]. The efficacy of PPIs in acid‐related disorders (e.g., GERD and *H. pylori* eradication) depends on the *CYP2C19* genotype. P‐CABs do not present this limitation [22]. Notably, vonoprazan provides similar acid suppression in Asians and Caucasians, and fexuprazan has similar pharmacokinetic and pharmacodynamic profiles in Korean, Japanese, and Caucasian populations [23, 24].

Administration of tegoprazan 50 mg for 8 weeks has shown effects comparable to those of esomeprazole 40 mg in terms of healing EE [12]. Considering the rapid and competitive inhibition of proton pumps with the first dose of P‐CABs [9, 25], we hypothesized that tegoprazan is superior to lansoprazole in terms of mucosal healing of EE even at week 2; thus, we aimed to confirm this hypothesis by performing a pilot study. However, we observed that the 2‐week mucosal healing rate of tegoprazan was only comparable to that of lansoprazole (Table 2). As P‐CABs exhibit rapid and stable inhibition of gastric acid secretion with the first dose [10], possible explanations for these negative results are necessary. Of note, even if a P‐CAB rather than a PPI is administered, 2 weeks may be too short a duration for complete esophageal mucosal healing. However, vonoprazan has previously shown a significantly higher mucosal healing rate than lansoprazole did even at 2 weeks (90.7% vs. 81.9%, *p* = 0.0132) [10]. Importantly, the proportion of subjects with severe EE in this Japanese study was 36.2%, which was higher than that in the present study (19/218, 8.7%). According to a recent meta‐analysis, the mucosal healing rates of EE between PPI and P‐CABs may not differ in cases of mild EE [26]. In this study, the lansoprazole group also showed a healing rate of 86.2% after 4 weeks of treatment (Table 2). Notably, in patients with the mildest EE (LA grade A), the healing rate was 89.1%, indicating that the group had almost reached its maximum healing rate after 4 weeks (Figure 2B).

PPIs have been reported to have unsatisfactory mucosal healing rates in patients with severe esophagitis (LA grade C or D) after 8‐week treatment [27, 28]. In contrast, our study showed that tegoprazan resulted in a higher healing rate than lansoprazole in patients with severe esophagitis (88.9% vs. 70.0% up to week 4, *p* = 0.582, Figure 2C). According to a meta‐analysis that included six eligible randomized controlled trials (RCTs), vonoprazan was non‐inferior to PPIs in terms of their efficacy and safety as therapy for patients with erosive GERD (RR [95% CI]: 1.06 [0.99–1.13]) but more effective than PPIs for patients with severe EE (1.14 [1.06–1.22]) [29]. In addition, a recent RCT showed that vonoprazan achieved significantly higher mucosal healing rates than PPIs in patients with severe EE; this finding was also comparable with the findings of the present study [30]. However, owing to the small number of patients with severe EE in this study, statistical significance could not be achieved. As current research on tegoprazan efficacy in treating severe EE is limited, more studies are needed to provide additional evidence for its effectiveness in this subgroup.

We must also determine whether short‐term treatment leads to better healing. For instance, PPI therapy may promote the microscopic healing of the mucosa and restore its integrity [31], which may not be visible during white‐light endoscopy. If the treatment duration is insufficient, mucosal integrity may not be fully restored even if EE is healed.

Regarding GERD symptoms, tegoprazan displays rapid onset of antisecretory activity and suppresses nocturnal acidity quicker than vonoprazan does [32]. In this study, however, the difference in heartburn‐free days between tegoprazan and lansoprazole did not reach statistical significance (Figure 3). This may be attributed to a type II error such as a small sample size. Moreover, as asymptomatic EE patients were also eligible to participate in this study, the secondary outcomes of symptom improvement did not yield significant results (Table S1). Nevertheless, the percentage of complete resolution in heartburn symptoms for tegoprazan was 52.9% from the first day of administration, which was equivalent to that for lansoprazole on the fourth day (59.6%, Figure 3A). In a recent RCT, tegoprazan in patients with non‐erosive reflux disease (NERD) showed a significantly greater alleviation of heartburn symptoms compared to the placebo [33]. These findings were also comparable to a previous small clinical trial of vonoprazan [11]. Considering the action mechanism of P‐CABs, these findings can be advantageous in shortening the treatment period and determining treatment effectiveness of acid‐suppressive drugs in patients with GERD [9].

This study had several limitations. First, this study was designed as a non‐inferiority trial; therefore, the sample size was relatively small. Although tegoprazan appeared to be associated with better outcomes, no statistically significant results were observed in the subgroup analyses. Second, the proportion of patients with severe EE (LA grade C or D) was low in this study population, with more than 50% of the enrolled patients having LA grade A reflux esophagitis, which is classified as “borderline or inconclusive” evidence for GERD according to the recent Lyon Consensus 2.0 guidelines [34]. However, the prevalence of GERD is increasing in Asia, but most Korean patients with GERD have NERD or mild esophagitis LA grade A or B, whereas the proportion of patients with LA grade C or D and Barrett's esophagus is very low [35]. In addition, some experts in Asia have different opinions on whether the same standards based on the Lyon Consensus guidelines should be applied to the Asian population [13, 36, 37]. To address the limitations, future studies will need to increase the proportion of patients with severe EE. Third, serum gastrin levels after 2‐ and 4‐week treatment with tegoprazan or lansoprazole were not measured. However, previous studies have already shown that, despite possessing similar antisecretory properties, tegoprazan causes less hypergastrinemia than vonoprazan [38, 39, 40]. Fourth, liver function was not investigated with a full panel, including the liver‐specific microRNA‐122 [41]. Nevertheless, previous studies have demonstrated that tegoprazan is safe in terms of hepatotoxicity. Recently, a Korean claim dataset‐based cohort study showed that tegoprazan had significantly lesser hepatotoxicity than other PPIs [42]. Fifth, the study should have lasted 8 weeks to confirm whether the 4‐week healing rate of tegoprazan is comparable to its 8‐week healing rate. However, this would require study participants to undergo multiple endoscopic procedures (four endoscopies in 8 weeks), potentially making it difficult to recruit participants. Finally, in this study, the superiority of tegoprazan over lansoprazole was observed in the FAS up to week 4; however, only non‐inferiority was confirmed in the PPS. Notably, the results of FAS and PPS appear to diverge at week 2. This discrepancy is attributed to the fact that, within the lansoprazole group, 12 out of 19 patients who did not achieve mucosal healing at week 2 withdrew their consent before the 4‐week follow‐up; in contrast, within the tegoprazan group, only 2 out of 12 patients who did not achieve mucosal healing at week 2 withdrew their consent. Although a high dropout rate was anticipated because of the requirement for three consecutive endoscopic examinations within 4 weeks, identifying the cause of the different withdrawal rates between the groups in this randomized study is challenging. Therefore, follow‐up of the results through future clinical trials is necessary.

Nevertheless, this study was the first clinical trial to show the 2‐week cure rate of tegoprazan and the first study to have a 4‐week treatment outcome as the primary endpoint in Korean patients with EE. In this study, 4‐week treatment with tegoprazan was effective as the initial treatment in patients with EE exhibiting clinical implications for the possibility of 4‐week treatment even in patients with severe EE. Recently, the 2021 Japanese GERD guidelines introduced treatment with vonoprazan, a P‐CAB, as the sole recommended initial treatment for patients with severe EE [14].

In conclusion, we confirmed that once daily administration of tegoprazan 50 mg showed non‐inferior efficacy in healing EE and tolerability compared to lansoprazole 30 mg. Moreover, tegoprazan 50 mg was superior to lansoprazole 30 mg in the treatment of EE up to 4 weeks. Notably, tegoprazan 50 mg may be more effective in faster EE healing and symptom relief. Compared with lansoprazole, tegoprazan appeared to be more beneficial in patients with severe EE. However, a superiority trial is necessary to confirm our findings.

## Author Contributions

Guarantor of the article: Prof. Suck Chei Choi. Specific author contributions: Cheol Min Shin: investigation (equal); methodology (equal); project administration (equal); writing – original draft (lead); writing – review and editing (lead). Suck Chei Choi: conceptualization (lead); investigation (equal); methodology (equal); project administration (equal); writing – review and editing (equal). Jin Woong Cho: investigation (equal); methodology (equal); project administration (equal). Seung Young Kim: investigation (equal); methodology (equal); project administration (equal). Ok Jae Lee: investigation (equal); methodology (equal); project administration (equal). Do Hoon Kim: investigation (equal); methodology (equal); project administration (equal). Yu Kyung Cho: investigation (equal); methodology (equal); project administration (equal). Ju Yup Lee: investigation (equal); methodology (equal); project administration (equal). Sang Kil Lee: investigation (equal); methodology (equal); project administration (equal). Jeong Eun Shin: investigation (equal); methodology (equal); project administration (equal). Gwang Ha Kim: investigation (equal); methodology (equal); project administration (equal). Seon‐Young Park: investigation (equal); methodology (equal); project administration (equal). Su Jin Hong: investigation (equal); methodology (equal); project administration (equal). Hye‐Kyung Jung: investigation (equal); methodology (equal); project administration (equal). Sang Jin Lee: investigation (equal); methodology (equal); project administration (equal). Young Hoon Youn: investigation (equal); methodology (equal); project administration (equal). Seong Woo Jeon: investigation (equal); methodology (equal); project administration (equal). In Kyung Sung: investigation (equal); methodology (equal); project administration (equal). Moo In Park: investigation (equal); methodology (equal); project administration (equal). Oh Young Lee: investigation (equal); methodology (equal); project administration (equal). All authors read and approved the final manuscript and the authorship list. All authors fulfill the ICMJE criteria for authorship.

## Disclosure

Declaration of personal interests: Ah Rong Kim, Hye Won Kim, Kyu Hoon Gee, Hyun Wook Park, and Geun Seog Song are employees of HK inno.N Corp., Seoul, Korea.

## Ethics Statement

The clinical protocol was approved by the Institutional Review Boards of each institute and followed the principles of the Declaration of Helsinki. All participants signed an informed consent form before inclusion in the study.

## Conflicts of Interest

The authors declare no conflicts of interest.

## Supporting information


Data S1.

## Data Availability

The data that support the findings of this study are available from the corresponding author upon reasonable request.
