# Supplementary material for: Comparison of Tegoprazan and Lansoprazole in Patients With Erosive Esophagitis up to 4 Weeks: A Multi‐Center, Randomized, Double‐Blind, Active‐Comparator Phase 4 Trial
Source: Neurogastroenterol Motil. 2024 Nov 25;37(1):e14969. doi: 10.1111/nmo.14969 (PMC11650551; doi:10.1111/nmo.14969)
Supplement: Supplementary file 1 — Data S1. [file NMO-37-e14969-s001.pdf]

Comparison of Tegoprazan and Lansoprazole in Patients with Erosive Esophagitis up to 4 Weeks: A Multi-Center, Randomized, Double-Blind, Active-Comparator Phase 4 Trial

Cheol Min Shin, Suck Chei Choi, Jin Woong Cho, Seung Young Kim, Ok Jae Lee, Do Hoon Kim, Yu Kyung Cho, Ju Yup Lee, Sang Kil Lee, Jeong Eun Shin, Gwang Ha Kim, Seon-Young Park, Su Jin Hong, Hye-Kyung Jung, Sang Jin Lee, Young Hoon Youn, Seong Woo Jeon, In Kyung Sung, Moo In Park and Oh Young Lee

List of Supplementary Materials

Supplementary Figure S1. Scheme of the clinical trial

Supplementary Table S1. Summary of the RDQ analysis results (full-analysis set)

Supplementary Table S2. Summary of safety outcomes (safety-analysis set)

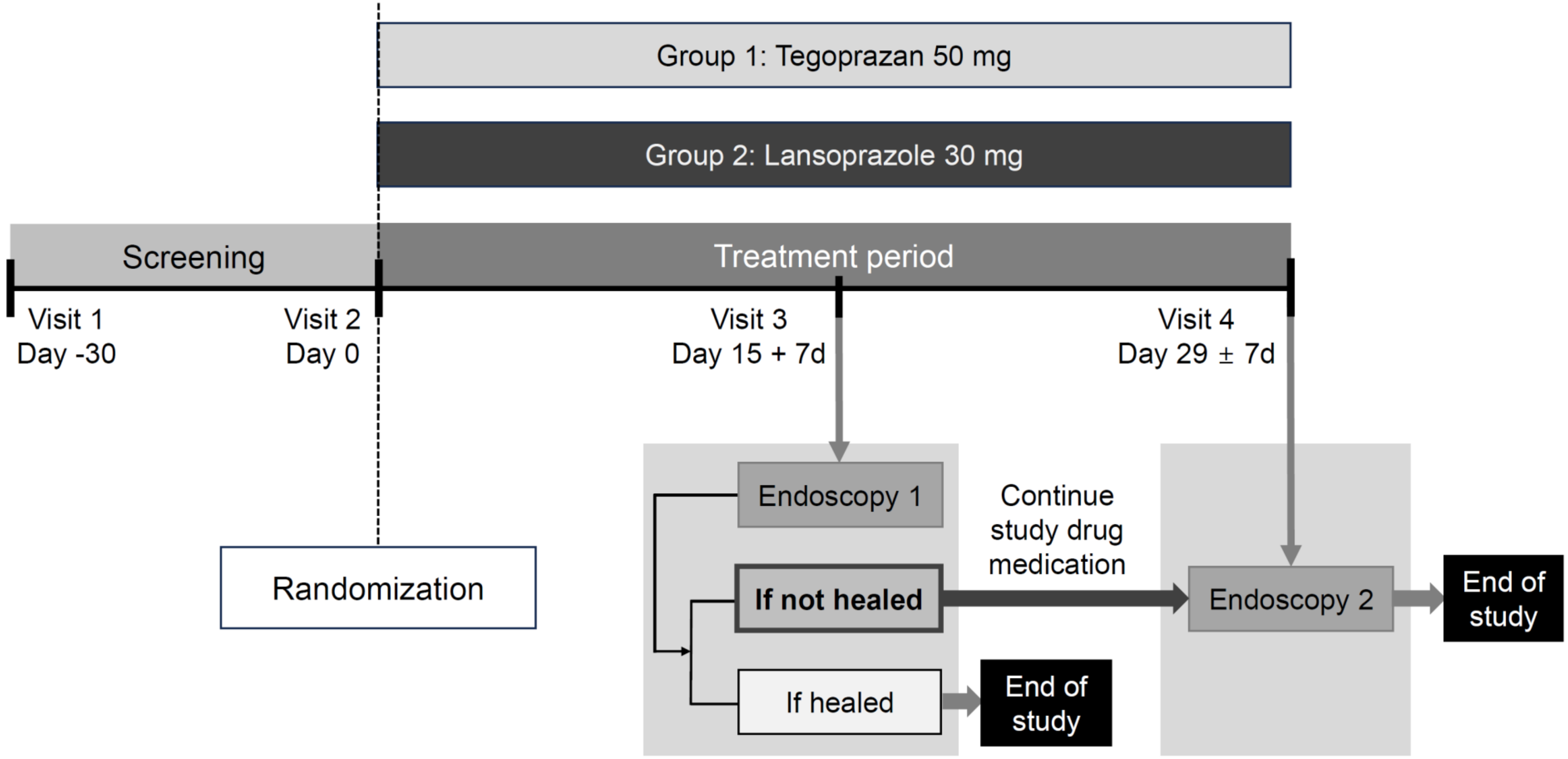

Supplementary Figure S1. Scheme of the clinical trial

Supplementary Table S1. Summary of the RDQ analysis results (full-analysis set)

	Heartburn			Dyspepsia			Regurgitation		
	Tegoprazan (n = 103)[a]	Lansoprazole (n = 109)	*P*-value	Tegoprazan (n = 103)[a]	Lansoprazole (n = 109)	*P*-value	Tegoprazan (n = 103)[a]	Lansoprazole (n = 109)	*P*-value
Change in severity score, mean (SD)									
Week 4	-1.95 (1.38)	-1.57 (1.37)	0.6157††	-1.25 (1.23)	-2.00 (1.41)	0.2968††	-1.45 (0.90)	-2.36 (1.35)	0.1143†
Week 2	-1.19 (1.14)	-1.01 (1.06)	0.3326††	-0.81 (1.07)	-1.08 (1.14)	0.0866††	-0.89(1.19)	-0.97 (1.17)	0.4708††
Change in frequency score, mean (SD)									
Week 4	-1.85 (1.00)	-1.57 (1.37)	0.6335†	-1.25 (1.14)	-2.21 (1.75)	0.1871†	-1.75 (1.06)	-2.36 (1.28)	0.3027†
Week 2	-1.13 (1.16)	-1.01 (1.18)	0.5002††	-0.74 (1.22)	-0.94 (1.10)	0.2228††	-0.84 (1.21)	-0.92 (1.39)	0.8269††

Data are shown as median (interquartile range). RDQ, reflux disease questionnaire; SD, standard deviation.
[a]6 patients in the tegoprazan 50 mg group were excluded due to missing results
†Unpaired t-test, ††Wilcoxon's rank-sum test

Supplementary Table S2. Summary of safety outcomes (safety-analysis set)

	Tegoprazan 50 mg (n = 108)*			Lansoprazole 30 mg (n = 109)		
	n	(%)	[F]	n	(%)	[F]
All TEAEs	7	(6.48)	[10]	3	(2.75)	[7]
Drug-related	0	(0.00)	[0]	0	(0.00)	[0]
Not related	7	(6.48)	[10]	3	(2.75)	[7]
Mild	5	(4.63)	[6]	3	(2.75)	[7]
Moderate	2	(1.85)	[4]	0	(0.00)	[0]
Severe	0	(0.00)	[0]	0	(0.00)	[0]
Leading to discontinuation	0	(0.00)	[0]	0	(0.00)	[0]
SAEs	0	(0.00)	[0]	0	(0.00)	[0]
Most frequently reported TEAEs by organ class and preferred term						
Nervous system disorders	2	(1.85)	[2]	1	(0.92)	[1]
Headache	2	(1.85)	[2]	1	(0.92)	[1]
Investigation	1	(0.93)	[1]	0	(0.00)	[0]
Increased alanine aminotransferase	1	(0.93)	[1]	0	(0.00)	[0]
Increased aspartate aminotransferase	1	(0.93)	[1]	0	(0.00)	[0]
Gastrointestinal disorders	1	(0.93)	[1]	2	(1.83)	[3]
Abdominal discomfort	0	(0.00)	[0]	1	(0.92)	[1]
Dyspepsia	0	(0.00)	[0]	1	(0.92)	[1]
Erosive gastritis	1	(0.93)	[1]	0	(0.00)	[0]
Nausea	0	(0.00)	[0]	1	(0.92)	[1]
Deaths	0	(0.00)	[0]	0	(0.00)	[0]

Abbreviations: [F], Frequency of TEAES; SAEs, serious adverse events.
*1 study participant who was assigned to the tegoprazan arm withdrew consent before taking the study drug and was excluded from the safety-analysis set.
